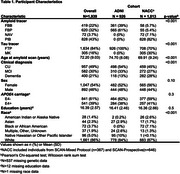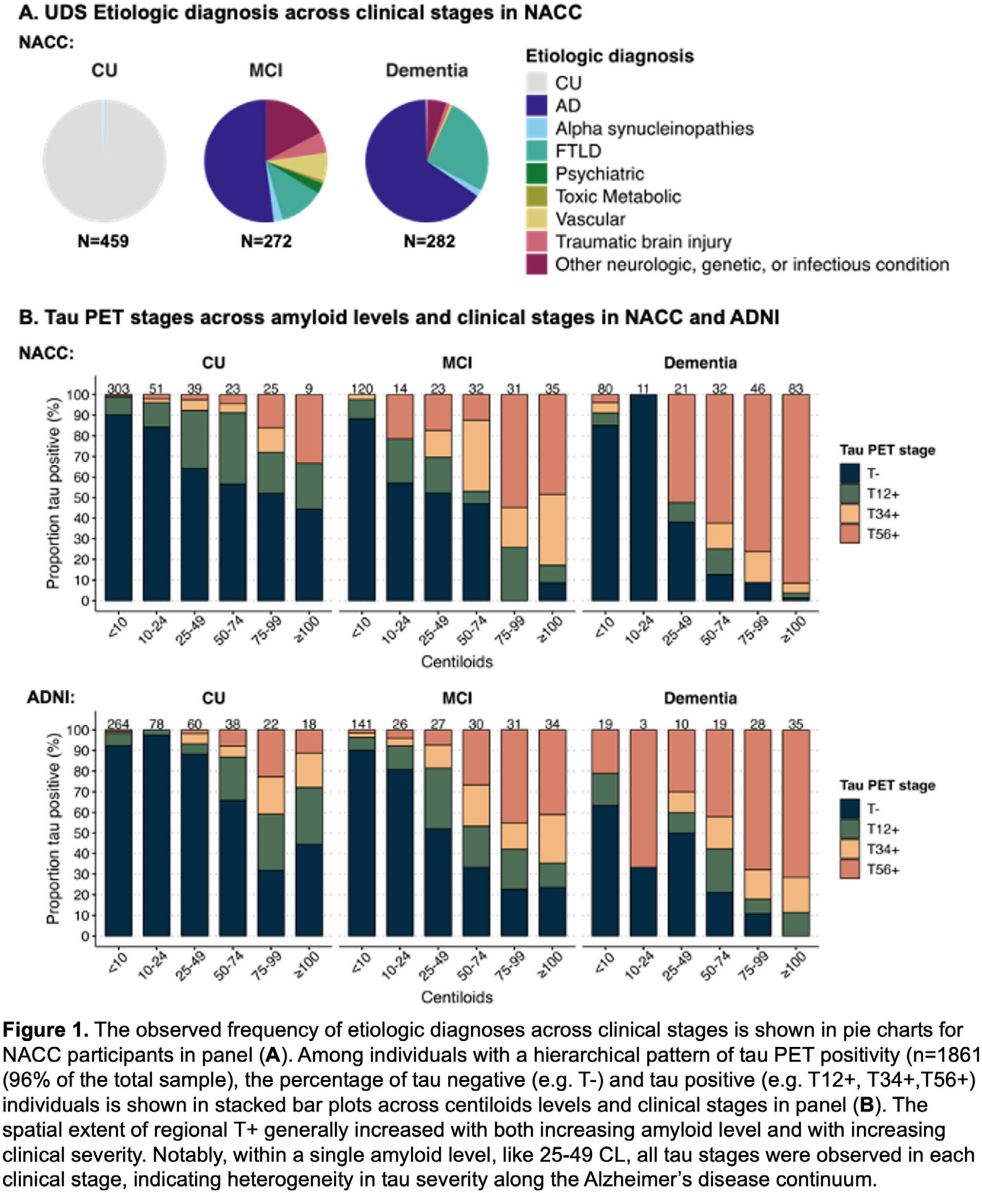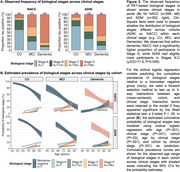# Characterizing amyloid and tau PET‐based biological stages along the clinical continuum in NACC participants

**DOI:** 10.1002/alz70856_104202

**Published:** 2025-12-26

**Authors:** Karly Cody, Andrzej Sokolowski, Joseph R. Winer, Christina B. Young, Kyan Younes, Alaina Durant, Logan Dumitrescu, Derek Archer, Duygu Tosun, Sterling C Johnson, Theresa M. Harrison, Timothy J. Hohman, Elizabeth C. Mormino

**Affiliations:** ^1^ Department of Neurology and Neurological Sciences, Stanford University, Stanford, CA, USA; ^2^ Department of Neurology and Neurological Sciences, Stanford University School of Medicine, Stanford, CA, USA; ^3^ Vanderbilt University Medical Center, Nashville, TN, USA; ^4^ Vanderbilt Memory & Alzheimer's Center, Vanderbilt University Medical Center, Nashville, TN, USA; ^5^ Department of Radiology and Biomedical Imaging, University of California, San Francisco, San Francisco, CA, USA; ^6^ University of Wisconsin‐Madison, Madison, WI, USA; ^7^ University of California, Berkeley, Berkeley, CA, USA; ^8^ Vanderbilt Memory and Alzheimer's Center, Vanderbilt University School of Medicine, Nashville, TN, USA

## Abstract

**Background:**

This study characterizes the frequency of amyloid and tau PET‐based stages along the clinical continuum in NACC participants from the Alzheimer's Disease Research Center (ADRC) program.

**Method:**

1013 NACC participants with available amyloid and tau PET imaging and diagnosis data were included in this study. Given the clinical heterogeneity present across NACC, parallel analyses were conducted in ADNI for comparison (*n* = 926; Table 1). PET data corresponding to ligand‐specific post‐injection windows were processed and harmonized across cohorts using an MRI‐free pipeline. Amyloid PET SUVRs were translated to centiloids (CLs). Amyloid positivity (A+/‐) was defined as 25CL. Regional tau positivity (T+/‐) was derived within ligand using Gaussian mixture modeling. A hierarchical staging schema was used to classify tau positivity (e.g., T‐, T12+, T34+, T56+) and create PET‐based biological stages (e.g., Stage A: A+T‐; Stage B: A+T12+; Stage C: A+T34+; Stage D: A+T56+). The frequency of PET‐based stages was examined across clinical stages and compared between cohorts. Ordinal logistic regression models were used to estimate the cumulative prevalence of biological stages according to age (mean‐centered), cohort, and clinical stage.

**Result:**

Compared to ADNI, NACC had twice as many individuals with dementia (Table 1), of which 35% were presumed to be due to non‐AD suspected etiologies (Figure 1A). Across cohorts, tau PET stages generally increased with both amyloid and clinical severity (Figure 1B). Notably, the frequency of biological stages within dementia differed significantly between cohorts such that NACC had a significantly higher proportion of participants in Stage D, while ADNI had more participants in Stages A‐C (χ^2^(3)=11.5, *p* = 0.009; Figure 2A). Analyses estimating the prevalence of biological stages reflected this difference in the distribution of biological stages across clinical stages between cohorts (cohort×clinical stage, *p* <.001). The estimated prevalence of the biological stages also differed by age and clinical stage (age×clinical stage, *p* <.001), with younger age associated with higher probability of Stage D in dementia across both cohorts (Figure 2B).

**Conclusion:**

Together, these findings underscore heterogeneity in amyloid and tau PET burden along the clinical continuum and highlight the application of amyloid and tau PET‐based staging frameworks in a clinically heterogeneous sample.